# Qualitative Content and Discourse Analysis Comparing the Current Consent Systems for Deceased Organ Donation in Spain and England

**DOI:** 10.3389/ti.2024.12533

**Published:** 2024-07-04

**Authors:** Kate Rees, Leah Mclaughlin, David Paredes-Zapata, Cathy Miller, Nicholas Mays, Jane Noyes

**Affiliations:** ^1^ NHS England, London, United Kingdom; ^2^ School of Medical and Health Sciences, Bangor University, Bangor, United Kingdom; ^3^ Donation and Transplant Coordination Section, Hospital Clinic, Barcelona, Spain; ^4^ Surgical Department, University of Barcelona, Barcelona, Spain; ^5^ Donation and Transplantation Institute Foundation, Barcelona, Spain; ^6^ NHS Blood and Transplant, Watford, United Kingdom; ^7^ Policy Innovation and Evaluation Research Unit, London School of Hygiene and Tropical Medicine, London, United Kingdom

**Keywords:** organ donation, consent, England, Spain, opt-out

## Abstract

England switched to an opt-out system of consent in 2020 aiming to increase the number of organs available. Spain also operates an opt-out system yet has almost twice the organ donations per million population compared with England. We aimed to identify both differences and similarities in the consent policies, documents and procedures in deceased donation between the two countries using comparative qualitative content and discourse analysis. Spain had simpler, locally tailored documents, the time taken for families to review and process information may be shorter, there were more pathways leading to organ donation in Spain, and more robust legal protections for the decisions individuals made in life. The language in the Spanish documents was one of support and reassurance. Documents in England by comparison appeared confusing, since additions were designed to protect the NHS against risk and made to previous document versions to reflect the law change rather than being entirely recast. If England’s ambition is to achieve consent rates similar to Spain this analysis has highlighted opportunities that could strengthen the English system-by giving individuals’ decisions recorded on the organ donor register legal weight, alongside unifying and simplifying consent policies and procedures to support families and healthcare professionals.

## Graphical Abstract

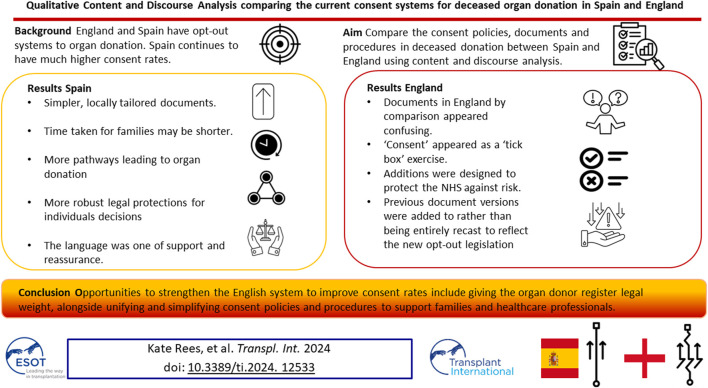

## Introduction

Since England switched to an opt-out consent system in May 2020, with the aim of making more organs available for transplant through the introduction of deemed consent, consent rates for deceased organ donation have not increased [1]. Despite high ambitions [2] England still appears to be falling short of the number of organs available achieved by Spain which has consistently had much higher organ donation rates (46.7 per million population in 2022) despite having a similar legal framework for deceased organ donation consent [3].

Presumed consent (sometimes referred to as deemed consent) means that a person is considered to have no objection to donating their organs after death unless they have registered or informed someone close to them that they do not wish to do so. There have been many studies that have concluded that presumed consent alone does not explain the fluctuation in donation rates between countries [4]. Legislation, public knowledge and awareness of organ donation, donor availability and characteristics, religious beliefs, transplant service infrastructure and healthcare system capacity (e.g., in intensive care), all play a part in making more organs available for transplant, but their relative importance is unclear [5–8].

England implemented their opt-out system to increase the consent rate for deceased organ donation, the assumption being that more organs would become available for transplant. However the opt-out legislation was nested within the existing opt-in system, and despite the addition of the new deemed consent pathway, the failure to secure consent for deceased organ donation retrieval from those involved in end-of-life discussions is still widely regarded as the single most important obstacle to making more organs available for transplant in England [9].

The purpose of this analysis was to identify differences and similarities in consent policies and associated documents between England and Spain and to consider whether there are opportunities to further increase consent rates for deceased organ donation and improve current practice in England.

### Overall Context and Scope

This analysis was undertaken as part of a broader evaluation of the impact of opt-out in England on the organ donation system [10]. During the study it became clear that the processes involved in consent, in particular were lengthy, excessive and negatively impacted families in England [11]. Specialist staff involved in consent also felt the process to be excessive and burdensome [12]. The research team was aware of extensive research into gold standards in terms of pathways to organ donation, i.e., what should happen and by whom to achieve the desired outcomes (the so-called Spanish model) [13] but was unable to find examples (or research) of the consent documents used in practice in countries with opt-out systems, which are considered leaders in organ donation. We felt that as the main study was specifically commissioned to examine a policy that (in theory) shifts from a model of informed consent to a model of presumed consent it would be a worthwhile and interesting analysis to look more closely at the consent documents and associated policies and guidelines of the world’s leading country with an opt-out system and compare them.

## Materials and Methods

### Research Question

What are the differences in roles, processes, consent forms and practices between the Spanish and English systems of organ donation and how do any identified differences begin to explain the higher consent rates in Spain?

### Data Collection

We identified and obtained key policy and procedure documents and consent forms from the websites of the “Organizacion Nacional de Transplantes” (ONT) in Spain and NHS Blood and Transplant (NHSBT) in England. Documents published in Spanish were translated into English for analysis using the “TransPerfect” computer software. Table 1 lists the documents included in the analysis [14–22]. The documents were read, reread, compared and coded.

**TABLE 1:** Documents included in the analysis.

**Document**	**Description**	**Page length**
*From Spain ONT:*		
Private Sector Donation [14]	Framework Protocol for organ and tissue donation in the private sector	93
Exchange SS1 2396 [15]	The basis of the Quality and Safety Framework Program for the procurement and transplantation of human organs and exchange with other countries	9
National Consensus Document 2012 [16]	Describes the situation in 2012 of asystole donation in Spain and other countries and provides a number of recommendations for the development of new these features and/or to improve the effectiveness of existing programs	205
Quality Improvement Programme [17]	This report shows the results of an evaluation of the current organ donation and transplant process (year 2019)	27
Royal Decree 1723–2012 [18]	Regulates the activities of obtaining, clinical use and territorial coordination of human organs intended for transplantation and establishes quality and safety requirements. (The first Legal document)	34
Barcelona University Hospital Consent Form	The current consent Form used for donation at the Barcelona University Hospital	1
Catalonia Regional Consent Form	The current consent form used for donation in the Catalonia region	1
Virgin Del Rocio University Hospital Consent form	The current consent form used for donation in the Virgin Del Rocio University Hospital	2 (page 1 consent, page 2 revoking of consent)
Emergency Professionals and the process of Donation [19]	Recommendations/Guidelines for Emergency Clinicians with respect to organ donation at presentation to hospital	27
*From England NHSBT:*		
Organ and/or Tissue Donation Manual (SOP5818/2) [20]	The guidelines governing organ and tissue donation within the United Kingdom	33
Code F: Donation of Solid Organs and Tissue for Transplantation. Human Tissue Authority (HTA) [21]	Human tissue authority Codes of Practice	44
Consent Form for Organ and/or Tissue Donation [22]	The United Kingdom wide consent form for organ and tissue donation	7

**FIGURE 1:**
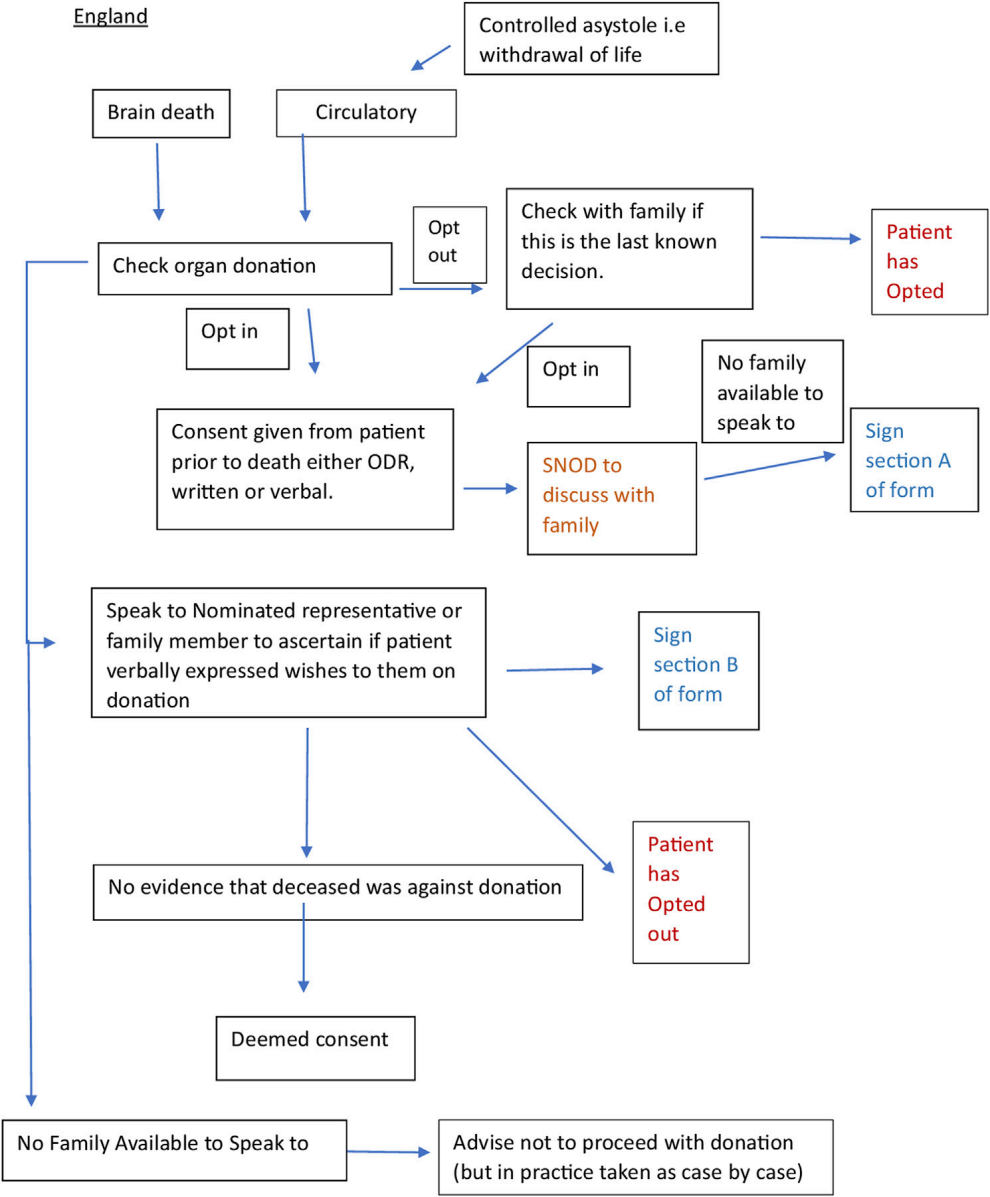
Flow chart of the English and Spanish process constructed from documents (20–22) and stakeholder engagement.

**FIGURE 2:**
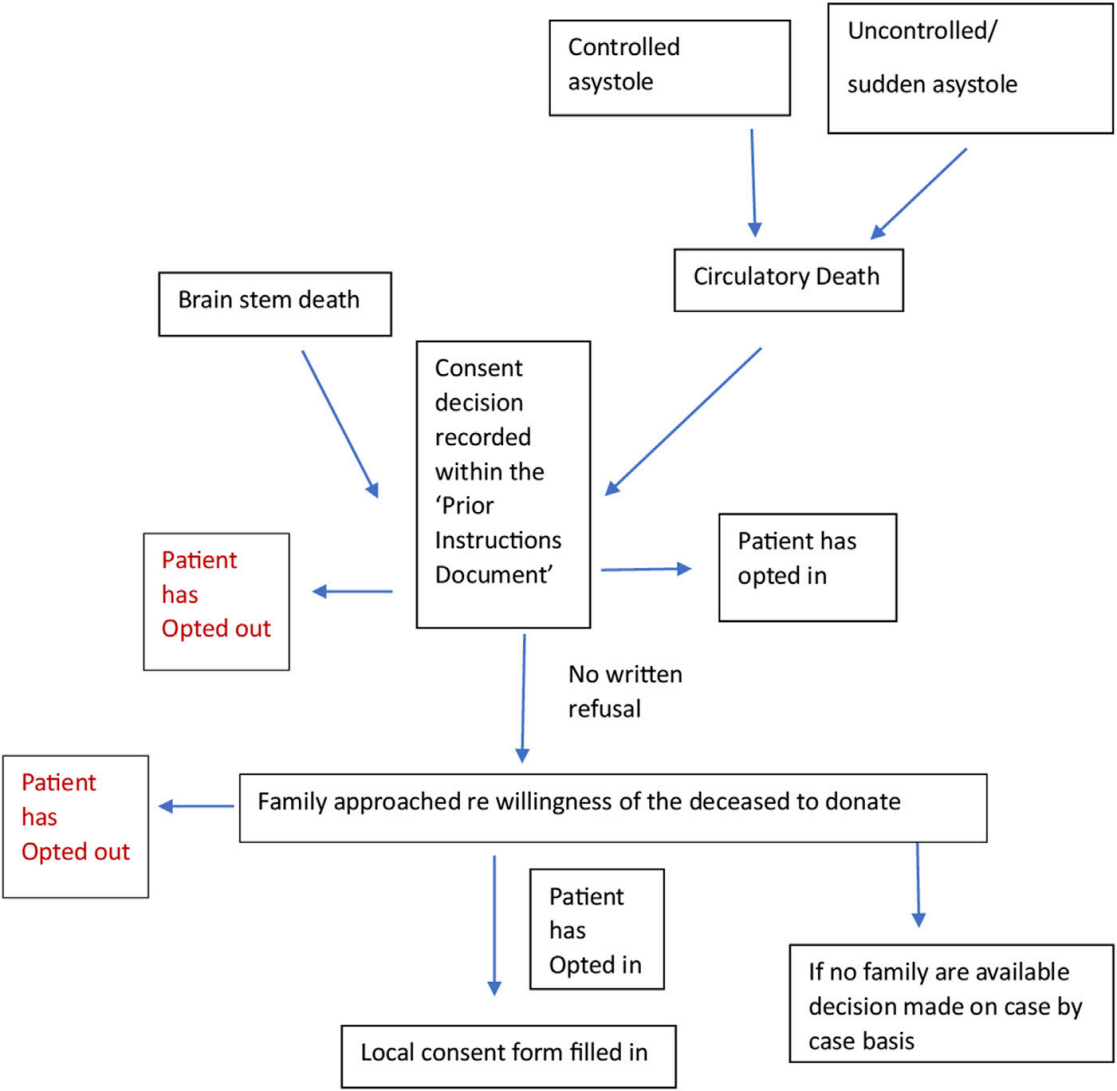
Flow chart of the Spanish process constructed from documents (14–19).

We worked with a Spanish intensive care doctor (co-author) via email and two online team sessions to clarify the correct interpretation of the documents and the donation system. This enabled us to verify that the current practice was in line with the written protocols. We engaged stakeholders through meetings with academics and cliniciansorganized by the European Society for Organ Transplantation (ESOT) to help establish the context of the English and Spanish organ donation systems within which the documents for analysis were produced. We consulted with a United Kingdom senior nurse (co-author) involved in the English NHSBT education program and United Kingdom legislation. A summary flowchart of the Spanish and English organ donation structures and processes was made for comparison (Figures 1, 2).

These processes helped to build a better understanding of broad cultural factors, such as religious beliefs, ethnic diversity, family dynamics, the reaction of families to the system and whether they had ever challenged the law, and how these might be underpinning any differences observed in the documents analyzed in detail.

### Data Analysis

Qualitative content analysis was used to code, analyze, compare and interpret the textual data and diagrams in the included documents to gain insight into the meaning and context of the policy, and the links between content, process and outcome [23].

Coding involved assigning attributes to words, sentences, or paragraphs to compare and contrast content, process and meaning. Consent forms were compared for structure, content, and length [24].

Principles of critical discourse analysis were used to make additional interpretations of the text, complemented by engagement with experts in the Spanish and English systems. This was done to systematically explore the often-opaque relationships between what is written (i.e., policies, guidelines) and what happens in practice, with multiple stakeholders, many with different objectives. This process helped to examine, for example, who or what the subjects and objects are in the respective structures, discourses and processes, and how and why the two systems manage to generate and maintain different forms of language (rhetoric) [25]. The flowcharts constructed (Figures 1, 2) helped to show where objects in relation to consent such as the Organ Donor Register, the roles of the staff, e.g., clinicians, transplant coordinators, nursing staff and the role and hierarchy of the family, etc. fit together in a complex system. The rhetorical analysis specifically searched for opportunities to give or decline consent within the process. This enabled us to understand more about the mechanisms underpinning the Spanish consent pathway, and thus extrapolate findings that may be applicable, with adaptation, to England [26].

### Author Reflexivity

Co-authors LM and JN were already working with colleagues in the English system and were connected via multiple professional networks to clinical and academic colleagues in Spain. Once the lead investigators had a good understanding of the two systems, we had further discussions with the Spanish consultant and Senior United Kingdom nurse co-authors to validate the interpretation of the two systems. We presented this work at several multi-disciplinary meetings and events including the Deconstructing Donation Special Interest Group for additional critique and insight. We adapted the recommendations for rigor, transparency, evidence, and representation to present the results [27].

## Results

Box 1 provides a comparison of some key performance indicators in England and Spain in the year 2022.

BOX 1 **Comparison of key performance indicators between Spain and England 2022.**

**Table:** 

**Key performance indicator**	**England**	**Spain**
Number of Transplants performed	51.3 per million population (pmp)	122.1 pmp
Deceased donor rate	20.1 pmp	46.7 pmp
Deceased donor consent rate	65%	84%

A direct comparison of the systems, processes, and cultural and linguistic styles between Spain and England in relation to consent for deceased organ donation is described below. Table 2 highlights similarities and differences within the systems with specific reference to consent (Table 2). The mechanisms that may or may not be a factor in achieving the desired outcomes in relation to consent are further unpacked and described in Table 3.

**TABLE 2:** Similarities and differences within the Spain and England systems with specific reference to consent.

	England	Spain
Consent system	“Soft” opt-out, opt-in and family consent for organs and tissues. Scheduled purposes and research not covered by the Act	“Hard” opt-out, opt-in and family consent based on the will of the deceased for scientific and therapeutic purposes
Eligibility criteria to apply opt-out system	Over 18, ordinarily (12 months prior to death) and voluntarily resident in England, dies in England, with full mental capacity	Over 18, has full mental capacity and be in adequate health
Age of consent for adults	18	18
Organs and Tissues included in opt-out system in place	Only organs and tissues “routinely collected and used for life saving/improvement treatments”	Includes both organs and tissues routinely collected for life saving/improvement treatments, scheduled purposes and research
Family made aware prior to admission to ICU to consider organ donation	no	yes
Family spoken to regarding withdrawal of treatment in DCD death and tests for DBD	yes	yes
Organ Donor Register	Yes – but has no legal status	No ODR in Spain
Prior Instructions Document	No	Yes – and has legal status
Determine the last known decision of the deceased	Yes	Yes
Nominated representative	Yes	No
Family hierarchy	Yes	Yes
Key hierarchical family member identified and spoken to as a priority	No (it is in the guidelines but rarely done in practice as a priority)	Yes
Witness to the conversation between SNOD and relatives/TC and relatives	Yes	Yes
Mandatory/legal requirement that family member signs donation form	No	Yes
Leaflets given	Yes – content and context varies	No
Details of all organs and Tissues taken explained	Yes	Simply
Details of body appearance following donation described to the family	Yes	Yes
Family continued to be supported by TC or SNOD if consent declined	No	Yes
Family follow-up	If signed consent given	No
Family informed of those whom donation helped	If signed consent given	No-can receive a thankyou letter if they sign for this
Can be contacted by those receiving donation	If signed consent given	No

**TABLE 3:** Mechanisms which may be a factor in bringing about the desired outcomes, or not, in relation to consent.

*Healthcare Professionals*	England Only SNODS/SRs are allowed to approach family members about organ donation. Anyone else is actively discouraged from mentioning organ donation. This is because it is thought that NHS staff may create a context where organ donation is not presented in an appropriate way leading to reduced opportunities to gain consent. During the family discussion the SNOD/SR guidance document suggests that SNODs should remain impartial but often the advice and legislation is open to interpretation, often misleading, with arguments for and against ways to act. Therefore interpretation of this depends on the individual SNOD/SR involved	Although all “*families are encouraged to support the decision their relative made in life*.” In England 43% of families said no in 2022–23 whereas around 10% of families still refuse in Spain (outcome). The Spanish system therefore contains more factors that create supportive contexts that bring about higher consent rates (mechanisms) Having a more unified and bespoke approach for the TCs and this being reflected in a wider culture of support appears to be a factor that creates a mechanism for achieving higher consent rates (outcome) In the Spanish system, the potential for organ donation can create a context that subsequently influences the decision as to which hospital the patient is brought, enabling discussions to occur about admittance to ICU purely for donation, rather than recovery (mechanism). By empowering those outside of ICU to consider organ donation, creates a context which helps highlight potential donors to the TC and potentially aid conversations to patients prior to their death (mechanism)
	Spain Although TCs are encouraged to speak to families about organ donation, other health professionals are able to offer encouragement for donation should it be mentioned earlier [19]. Organ donation is thought of by health professionals outside of ICU and thus a lot earlier in the care pathway of the patient, even extending to community and emergency services	
System configuration	England ICU beds remain a scarce and precious resource to treat patients who are alive. There are no specialist organ donation centres in England. Every acute hospital is able to offer/honour organ donation on site as it is the organ retrieval team and SNOD/SRs who travel to the hospital	The lack of ability to admit potential organ donors to ICU purely for organ donation reflects unequal End of Life care policies between England and Spain (comparative context) and could help explain the differences in consent rates (outcomes) but also potentially indicates a discrepancy in priorities between countries (contexts and mechanism) that also impacts negatively on consent rates (outcome) In Spain organ donation is more visible and acceptable – due to capacity to host more potential organ donors without adding strain or worry to the ICU service. This creates a context and mechanism that makes organ donation easier. The NHS would however need to increase ICU capacity to adopt this approach to create a similar context and mechanism leading to better consent outcomes In both countries it is specialist teams that provide the care (context), but in England the more complex process can take hours to days (context). This means that the family may have to wait a length of time before being able to speak to the SNOD/SR and go through the longer processes (mechanism) and this can often influence their decision to decline donation (outcome) The length of time can also give families more time to revoke consent (mechanism leading to outcome) if it is given and they may decline consent straight away feeling that their loved one has already suffered enough or to be able to start making funeral arrangements (mechanism and outcome)
	Spain Patients in Spain can be admitted to ICU purely for the purpose of donation. Spain has specialist organ donation hospitals which have designated TCs	
*Faith and Beliefs*	England Throughout the English guidelines faith/beliefs are mentioned frequently and there are documents dedicated to this. There is also the option of recording this when someone registers a decision on the organ donor register	While there are detailed guidance on faith/beliefs (context) the guidance in the documents for healthcare professionals and options on the ODR are not translating into practice - vast inequalities remain in organ donation in the United Kingdom. (outcome)
	Spain Although faith and beliefs are important they are rarely specifically mentioned in the documents or given a huge amount of coverage	
*The organ donor register*	England The organ donor register enables people to record a decision about organ donation prior to death. It enables people to choose which organs and tissues they would like to donate or not. However there are many avenues to recording a decision, the forms are not universal and they do not reflect what the family is asked after death. Therefore despite people making these decisions the family will still be approached and questioned to ensure that the decisions made by the potential donor have not changed In England the HTA states that although “*consent has been obtained, it is not mandatory that organ donation proceeds”especially if “the family do not support it.”* The SNOD/SRs are left to determine each situation on their own best judgements as the current guidelines are not clear Ironically if the nominated representative cannot be contacted in time, consent can be deemed yet if no family are available and there is nothing recorded on the ODR it is advised that consent does not go ahead	In England, although the organ donor register gives the opportunity for people to record a decision prior to death it does not have any legal status (context). This means that family members can easily override their relative’s decision to donate their organs made in life (unintended mechanism) resulting in lowered consent rates than anticipated (outcome) In England, the consent process for the bereaved family is more burdensome (context), potentially contributing to revoking of consent or reluctancy to give consent (mechanism and outcome). It can also be a surprise to the family that a decision has been recorded by the potential donor as a decision can be made effortlessly when applying for a boots advantage card or drivers licences, for example, but these are kept separate and independent from medical notes (context and mechanism). This could help explain why the numbers of people opting-in to donation have increased somewhat, but overall consent to deceased organ donation has not (outcome) In England, deemed consent is not properly or always understood by family members yet as a positive decision that supports organ donation and perhaps why families are continuing to override the deemed consent
	Spain The patient will be required to get a form from their GP which has to be signed by a witness. This decision is then shared on their health record. Due to the increased effort in Spain to register an opt-out decision which is witnessed, this may explain why there are higher numbers of organ donation but also that families are more likely to discuss the decision with their relatives or friends and have more trust in the system that it is an integral part of end of life care In practice an opt-in decision is always discussed with the family, and the guidance advises that even opt-out decisions should be discussed to ensure this was the last known decision	
*Opportunities to say no*	England There are further opportunities for consent to be declined as highlighted in Figure 1. The potential donor can opt-out/in via the ODR or by expressing it verbally to family and friends. The nominated representative may also decline donation. By further checking if an opt-in decision on the ODR was the final decision offers a further opportunity. If the family disagree with the potential donors decision in life, sometimes donation does not occur out of fear of upsetting the family and risk to what messages would be interpreted by the wider public. The family are frequently reminded that they can decline at any point until the retrieval has commenced, *“Withdrawal of consent should be discussed at the outset when consent is being sought.”* This is also a regulatory requirement written into the procedural documents before deemed consent was introduced This suggests that no is the default answer expected, which is the opposite of a deemed consent system	Despite a law which switched the default to one where organ donation is presumed, documents and guidance appear to support the opposite in the England (context). Tailoring this part of the process to the family and being allowed to speak more simply about the organs, tissues and processes may make this process easier and shorter. Therefore easier to say yes, and easier for everyone involved to go through (mechanism) and give their consent to organ donation (outcome)
	Spain Consent to donation can be declined either by writing in the prior instructions document or declared to the family who can then continue to decline on the potential donors behalf after their death. In Spain the TC will strive to understand the reasons why donation is declined and they are encouraged to give the family time to ensure this is the final decision before accepting it	
*The family and language*	England When families are approached, they are asked what the potential donor’s last decision would have been and whether the deceased expressed any thoughts on becoming a donor. The current policy suggests that the family are approached according to the highest qualifying relationship. This does not always happen in practice as the SNOD/SR tries to navigate the family dynamics while at the same time tries to gain evidence to support a ’*final decision on donation’* that can be from any family member	Rhetorically, this language possibly evokes feelings and thoughts of being brave and confident in testing times (context and mechanism). In England, the language appears to be less emotive by asking about the last known decision of the potential donor, which may not be as impactful as the rhetorical language typically used and encouraged in Spain Willingness itself evokes feelings or thoughts about the inclination or desire to help others if it is needed (context and mechanism). It appears to be almost a leading question. Nonetheless family dynamics can often be difficult to grasp and work with, particularly at times of acute grief. Families are complex and not all respond in the same ways to simple linguistic interventions and again this mechanism does not always work in practice (outcome)
	Spain The family are asked what would have been the willingness of the deceased to donate their organs and the key family member is identified In both countries the family are made aware that donation can be declined (if no decision has already been made by the deceased). In both cases the decision is respected and the TC/SNOD seeks to understand why However, in Spain the TC gives the family time to further think about their decision before accepting it as the final decision. The TC can also bring up ethical arguments for organ donation and also use the argument that it is likely that they will need an organ in their lifetime, which could influence the family decision and use arguments for courage, generosity and proximity, e.g., *“you are likely to need an organ at some point in your life*”	
Consent forms	England The 7 pages consist of yes/no tick box answers for a list of organ, tissues and processes involved in donation. For every donation (even for opt-in decisions on the ODR) the family will go through the same process. This is done to conform with the human tissue act 2004, although the forms have no legal status and are not mandatory to sign	The length and detail of the consent process and form (context) could become overwhelming for a family and dissuade them from supporting (mechanism) the current donation (outcome) or what they perceive might be involved in the future in terms of retrieval (mechanism). The consent form may also leave SNODs/SRs feeling vulnerable given it is not mandatory for the authoriser to sign especially if there has been some conflict on the final decision between the family (context and mechanism), and the SNOD/SR more likely to stand down (outcome). The SNOD/SR may also be more likely to accept a decline in consent (outcome) given the mixed messages in the legislation and guidance, and if the family are divided, or especially traumatised, or the donor is borderline given the additional time and burden of the consent and retrieval processes (mechanism)
	Spain Consent forms are created by local hospitals using the ONT template. They are short (one/two pages) and it is mandatory for the authorising family member to sign. Some forms have space for the family to write which organs they believe donor would or would not wish to donate; in others this decision is written within the medical notes instead. The form has legal status	

### Overall System

England has a diverse population with deep-rooted Christian traditions and multi-faith communities. England switched to an opt-out system of consent to deceased organ donation in May 2020. The organ donation system is run by the NHSBT, which is publicly funded and not privately available. Deceased organ donation is considered for those who die from brain stem or controlled circulatory death. Donation is therefore only possible for those who are admitted to an intensive care unit (ICU), but ICU admission is for treatment and prognostic purposes, not for organ donation [28–30].

England has an intensive care bed capacity of approximately 6.6 per 100,000 people [31]. Organ donation is possible in every acute NHS hospital. When the patient is identified as a potential donor the clinical team caring for the patient will refer the patient via a national referral number. The regional NHSBT team will assess the patient and mobilize a Specialist Requester (SR) or Specialist Nurse in Organ Donation (SNOD) – depending on who is available. After checking the national Organ Donor Register (ODR), the SNOD/SR will visit the unit and approach the family about donation – this is a nurse-led process and care pathway. The ODR has various options (e.g., opt-in, opt-out, nominate a representative and the ability to specify a small number of organs/tissues that people do or do not want to donate after death) but it has no legal status and family members have the ability to override it in practice and even register a decision on behalf of their loved one. Hospitals are reimbursed a small sum for facilitating organ donation, (approximately 1,000 pounds per donor) but this figure has not increased substantially over time and complex and bureaucratic finance systems often make it difficult to spend and save money to promote organ donation.

Spain is a predominantly Catholic country and has had an organ donation system for 44 years [32]. The organ donation system is overseen by the ONT. It is possible to be an organ donor while being treated privately, by being transferred to the public health system for donation purposes only. In addition to the pathways in England, deceased organ donation can be obtained from sudden unexpected circulatory deaths and those undergoing euthanasia. Spain has an intensive care bed capacity of approximately 9.7 beds per 100,000 people [33]. In Spain, patients admitted to the Emergency Department with catastrophic brain or cardiac damage where treatment is considered futile, can be intubated, and admitted to the ICU for the purpose of organ donation [34]. Also, those who are suspected of developing brain death or have already been declared brain dead in private institutions or the Emergency Department, can be admitted to the ICU solely for the purpose of organ donation, unlike in England.

Spain has dedicated hospitals where deceased organ retrieval can occur, with designated transplant coordinators (TC) in each of these hospitals (approximately 70% being physicians and 30% nurses). Often in hospitals with no TC, there will be proactive ICU staff who can identify donors. They can request support from a dedicated hospital which will usually send a TC to aid in speaking with the family. Any healthcare professional can contact the TC regarding a potential donor. Once alerted to a potential donor the TC will visit the potential case, review the medical records, and determine whether or not there is a “*prior instructions document.*” This document has legal status.

### System Processes Concerning Deceased Organ Donation Consent

In England “*the individual leading the family approach for organ donation must be suitably trained and qualified with sufficient knowledge and skills to sensitively answer any questions and have the time to support the family,”* [21, pg 9]. In practice, this is always the SNOD/SR, anybody outside of this role is actively discouraged from discussing organ donation [12].

As illustrated in Figure 1, the English system has many pathways to consent. If the deceased opted for organ donation during their lifetime, this is discussed with the family to ensure that this was the last known decision. If the deceased had opted out of the ODR “*providing work load allows, the SNOD should also discuss with the family if this was the last known decision.”* [SIC] [20, pg 11]. If this is not possible due to workload, the SNOD/SR will “*coach the clinician in the discussion to have with the family and agree actions.”* If the clinician feels unable to do this, the family will have to wait for the arrival of the SNOD/SR. In practice, detailed discussions with the family when the deceased has opted out rarely happen due to limited resources and concerns about NHSBT being seen as pushing for organ donation when the deceased has opted out.

Another pathway, although rare, is the “nominated representative,” where a person nominates someone else to make a decision on their behalf before they die. “*If despite all reasonable efforts the nominated representative cannot be contacted in time or to make a decision, then consent may be deemed.*” [SIC] [21, pg 19] Nonetheless, the donation can only take place after the family has also been consulted.

Only after the SNOD/SR has established that none of the above pathways apply, can they check whether consent can be assumed. If the family cannot agree, despite being given time and further information, then “*the hierarchy of consent, i.e., highest qualifying relationship,” applies but the final decision to proceed lies with the [SNOD/SR].*’ [SIC] In reality, it is the family member with the strongest voice (either for or against donation) whose wishes are followed [11, 35]. In addition, the SNOD/SR cannot proceed with the donation unless they have the full support (and permission) of the treating clinical team(s). If the family cannot be contacted and there is no prior expression of a decision, then although *“consent could be deemed it is advised that donation must not proceed.’*”[SIC] [21, pg 17].

To override a decision, families need only provide a “*level of information that would lead a reasonable person to conclude that they [i.e., the deceased] did not want to be a donor.”* [21, pg 24]. This may be verbal or written. Any evidence from any family member at this point can be taken into account. [21, pg 18] The SNOD/SR will make a judgment about the reliability of the information and whether it is right for the donation to proceed. “*Sometimes clinical staff will reach the judgement that although there is a legal basis to proceed with the donation, the human considerations involved mean that it should not go ahead. While the presence of appropriate consent permits organ and tissue donation to take place, it does not mandate that it must….(and) where the risks to public confidence might outweigh the benefits of donation proceeding, donation should not proceed even though the law permits it.*” [SIC] [21, pg 7].

In Spain, there is no organ donor register but a prior instructions document is available from the patient’s GP. Patients can register their consent or refusal to be an organ donor in the document which will be made available in the local Advance Directives Registry. Their families will be approached and informed of the recorded decision. If a “No” to the donation has been recorded, the family will still be asked if there has been any recent change to this decision. However, there would have to be substantial evidence to overturn this notion since the prior instructions document has legal value and is signed by a witness.

It is recommended that the healthcare professional who mentions organ donation be different from the professional who has discussed the likelihood of the patient dying to avoid a conflict of interest for the TC who may also have a role as an intensivist, etc. It is mandatory in some hospitals that the TC be contacted before withdrawal of treatment in the ICU, a condition introduced by some hospital medical directors.

### The Consent Forms

The English consent form is seven pages long, with all organs, tissues and retrieval processes listed as yes/no checkboxes, including options for additional information. The family will need to answer “Yes” or “No” to everything irrespective of what the deceased had registered about what organs they wanted to donate while they were alive and this will include organ donation for research (not just therapeutic purposes). The family will be made aware that the decision can be revoked until *“knife to skin.” [*20, pg 24]. The family members “*are encouraged to sign the consent form*” although there is no legal obligation to do so. The process may take hours to days. The SNOD/SR will document the conversation in the patient’s notes and on the NHSBT’s national digital system, also verified by a witness. If the family were to override the decision or revoke consent this will be respected and the reasons would be acknowledged and recorded by the SNOD/SR.

Each Spanish region has its own form based on examples from the ONT protocols. Often they are a single page requesting the name and relationship of the relative and the date. Some do have a free text space for the family to write what organs or tissues they believe the deceased would not have wished to donate. In other cases, these wishes are documented in the medical notes instead. Once a decision is reached after discussion with the family, it is mandatory that the consent form be signed by the dissenting family member(s).

### Approach and Language for Consent

In England, when families are approached, they are asked, “*what the potential donor’s last decision would have been and whether the deceased expressed any thoughts on becoming a donor” [SIC].* The guidelines suggest that SNODs/SRs should establish who is the next of kin (in accordance with the established highest qualifying relationship guidance) and approach that relative to organ donation. Although the opportunity to help others is often mentioned, the overall guidelines suggest that the SNOD/SR should remain impartial [21].

In Spain, if there is no recorded decision made while alive, the family is generally asked: “*what would have been the willingness of the deceased to donate their organs to help other people?” [SIC] [*16, pg 197]. *“If the family are in doubt, the TC can assist in decision-making, reinforcing positive verbalisations to donation and courage in those moments, and conveying ideas of generosity and proximity and enquiring whether the deceased gave to charity or donated blood during their lifetime, etc.” [SIC]* [16, pg 126].

In the case of large families, the TC seeks to speak to the “key family” member. The key family member is identified through discussion with the family and the knowledge of the staff caring for the patient. Should a family be divided over the issue of donation, the TC will not proceed. If there is no family present, the TC *“strive(s) through links with social services and the police to find a family member”[16, pg 120]* but may still consider organ donation if no family can be found.

Should the family decline the donation, “*it is important to make it clear that the decision is respected and understood but that, however, it is advisable to think about the matter more slowly without the presence of a TC.”[16, pg 126]* The TC also explores the reasoning behind the refusal and corrects misunderstandings. The TC may approach the family as often as necessary.

During the consent process, the family is usually asked which organs they believe the deceased would not want to donate. The conversation aims to combine “*speed and effectiveness in communicating with families, with respect for ethical principles and transparency that must preside over the process*.” [SIC] [16, pg 116] On average, the process of gaining consent takes 30 min.

## Discussion

This is the first detailed documentary comparison between the Spanish and English opt-out systems of consent to organ donation. The biggest differences observed were that the Spanish system was less complex in terms of consent, evidently pro-donation with a willingness to take some risks, likely to take less time, better resourced, with better access to ICU beds and a more locally tailored opt-out system with some legal protection for the potential organ donor’s life choices. England in contrast has a more complex centralized system with risk-adverse protocols, an itemized approach to consent, implemented in a country where there are fewer ICU beds, and no legal protection for the potential organ donor.

The Spanish system covers both public and private hospitals and has dedicated resources for organ donation, such as stand-alone centers and in-hospital beds. In England, for deceased organ donation, the NHSBT only covers NHS hospitals so some potential donors in the private sector are lost. There are no dedicated resources in England organ donation takes place when the system has the capacity to manage it which can potentially lead to frustration and disengagement of non-specialist staff. Euthanasia and organ donation are legal in Spain (illegal in England) and although the pathway is relatively recent it has created an additional platform to embed organ donation as a routine end-of-life process–the initial requests for this pathway have come from people who had requested euthanasia and not in the originaleuthanasia protocols. Potential organ donors with neurodegenerative conditions requesting euthanasia also tend to be younger without underlying co-morbidities and a single donor could potentially decide to donate all of their organs and tissues to help others, again increasing the visibility of organ donation in the system.

Families are as involved in decision-making in Spain as they are in England, but the consent process is shorter in Spain. The language used with family members and staff was also observed to be different in tone and meaning. The English system focuses on establishing the “*last known decision of the deceased*”whereas the Spanish system aims to establish “*the will of the potential organ donor to donate their organs as well as the will to help others.”* In England, current guidelines and codes of conduct reflect the human tissue authority’s position on consent to organ and tissue retrieval. This appears to be more in line with the old “opt-in” system and thus encourages unnecessary risk aversion which is contrary to the spirit of the opt-out legislation and appears confusing and neutral.

Organ donation appears to be more embedded within the Spanish healthcare system as an integral part of end-of-life care, with many healthcare professionals being aware of it and being encouraged to be involved with it. As such, it may be more likely to be discussed by families as there may be a healthcare worker in the family or someone they know who has been through the process before.

The legally binding prior instructions document is also available from the GP or local hospital and is signed with a witness present. Therefore, the witness, i.e., an accompanying family member is likely to be able to verify the document. Once completed, it is part of the person’s local medical record, meaning that there is a more complex process if family members want to challenge their loved one’s organ donation decision in life. There is a significant risk to donor decisions in England as anybody can go onto the ODR and register - SNODs/SRs continue to find cases where the opt-out decision was registered at a time when the person was being ventilated in the ICU [9].

The structure of the hospitals - i.e., that specific hospitals manage deceased organ donation, that patients can be admitted to the ICU purely for the need to ventilate organs and drug infusion in preparation for donation - is also very different from England. Matching the Spanish approach would undoubtedly cost the NHS more at the expense of another area of the health service. However, Spain states that “*the social value of organ donation justifies staff efforts and the economic cost involved”* [SIC] [16, pg 195], indicating an overall difference in priority in terms of deceased organ donation between the two countries.

In addition to the marked differences in the provision of ICU beds required for organ donation to proceed, in 2019, Spain had 3 hospital beds per 1,000 people whereas England had 2.5 beds per 1,000 people. In 2019, Spain had a bed occupancy rate of 76%, whereas in England the same rate was 92% for general and acute overnight beds [33]. Given the relentless pressure on NHS staff to continuously manage such a high bed occupancy rate, it becomes clearer why a centralized system of organ donation was implemented via a separate NHS body (NHSBT) with its own governance and management structures [36]. The NHSBT was created in 2008 and to a certain extent, its centralized opt-in system was successful in that consent rates steadily increased over the following decade before the law was changed. Nonetheless, the NHSBT has not been able to replicate the success of Spain. In 2020, a “soft” opt-out was implemented within the existing centralized national system alongside the existing opt-in system, and the two systems have been operating together in a complex way ever since. Although Spain does also offer the ability for patients to opt-in through their decision on the prior instructions document, this is rarely seen since the Spanish public trusts the organ donation system and knows that their families will always be consulted so they do not see it as important to record their life decisions. This makes the law appear more consistent and in line with a system of presumed consent, unlike in England [37].

### Recommendations for Policy and Practice in England and the United Kingdom

The NHS is built on the ethos that “if it is not written down it did not happen.” This has been generally applied to mitigate any potential legal action against staff or the NHS in the future. This is partially why consent documents and protocols tend to reflect ambiguity and risk aversion when compared with Spain which appears more comfortable with the spirit of presumed consent. However this is potentially creating a context where SNODs/SRs are not able to openly and proactively emphasize the benefits of organ donation or feel fully supported to do so with families. We suggest, given a change in legislation that has changed the default for nearly 60 million citizens to support the donation of their organs after they die, unless they say otherwise, that documents and standard operating procedures, particularly in relation to consent, reflect this and are revised with a view to simplification and presumption.

The ODR also lacks legal status. Approximately 10% of families override their relatives’ opt-in decision but the same rates are not observed for opt-out decisions. Despite having an ODR, it is not mandatory to follow the organ donation decision on the register. If the ODR was given greater legal status and the decisions in it were used as a basis for the conversation with the family after death (preferably by simplifying the latter to bring it more in line with the Spanish approach to consent after death), this could make it easier for the family to support the potential donor’s decision. It may also create a context in which people are more likely to discuss what they want in terms of organ donation. Aligning language, processes and guidelines with the legislation on presumed consent may generate a more positive initial response to organ donation and help address doubts or concerns that are common in these complex end-of-life discussions.

The linking of the ODR to a patient’s medical record can also make it easier for healthcare professionals to discuss with the patient if they still stand by their recorded decision should anything life-threatening happen during their admission, similar to a “Do Not Attempt Resuscitation” form.

**FIGURE 3:**
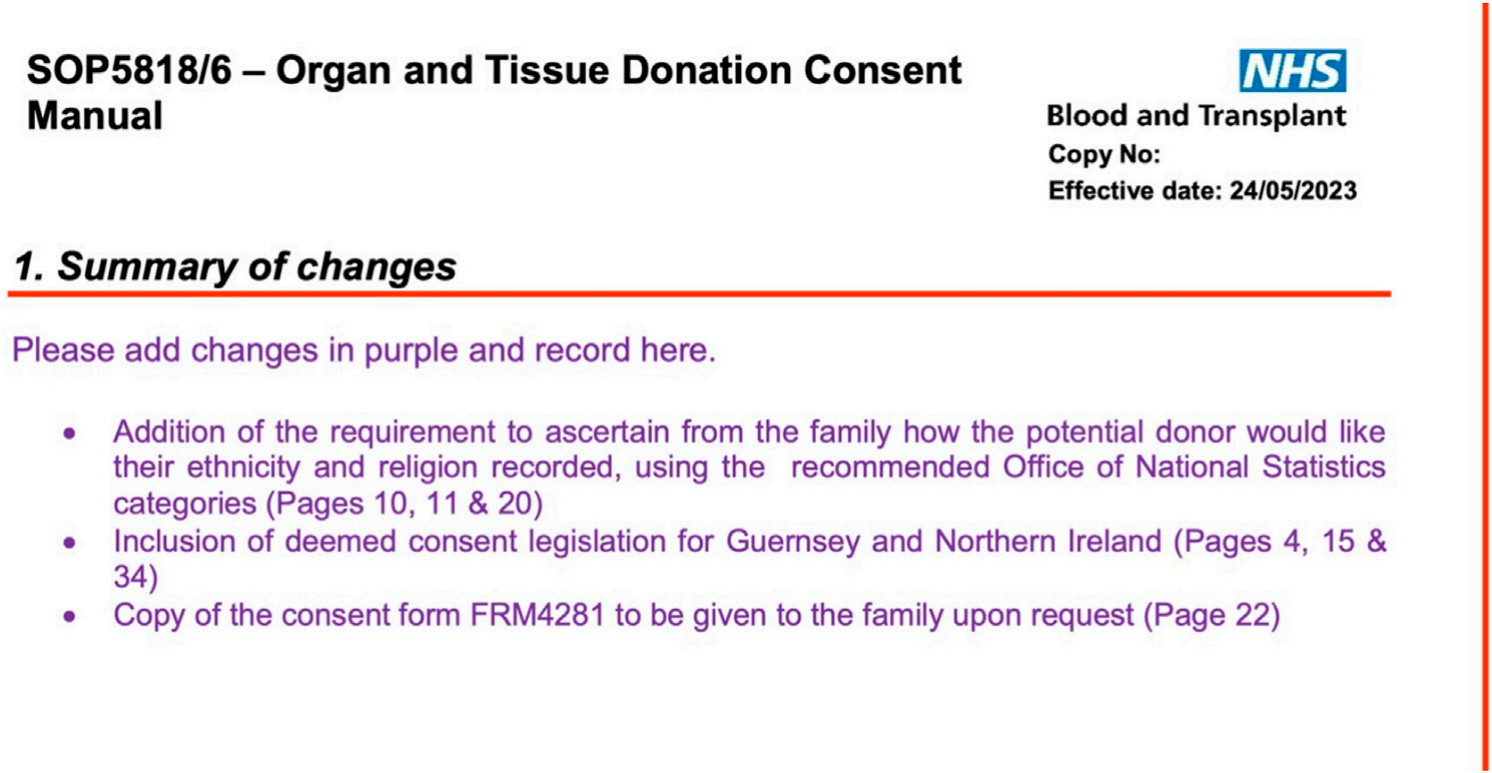
Latest update to the consent manual in England.

Although organ donation has expanded in Spain, the underlying principles of legal standing, guidelines and protocols have not changed substantially. Since 2021 the latest NHSBT consent manual has had six updates. The most recent updates are included in Figure 3 for reference. The consent form has undergone multiple revisions in recent years, with each iteration adding further layers of complexity and processes. This is wasteful and prevents opportunities for innovation that would benefit patients at an individual level. Any revisions to the documents need to be more mindful of the users (e.g., SNODs/SRs and acutely bereaved families) and provide a more personalized and sensitive approach to consent that is aligned with the ambitions of opt-out legislation.

### Limitations

Due to resource constraints, we were not able to back-translate very long policy documents from English into Spanish. We relied on software to translate Spanish documents and then verified key concepts and processes with a small number of Spanish experts.

Policy documents alone do not entirely reflect actual practice and there is of course variation in the implementation of processes within each health system. We acknowledge this limitation and mitigated it by engaging with organ donation practitioners in Spain and England as co-authors to complement our documentary analysis with their perceptions, experiences and knowledge. There are also significant differences within and between countries that are not reflected in a discourse analysis focused on consent such as detailed public attitudes to organ donation [37, 38] as well as potential donor characteristics and methods of optimizing organ donor potential which vary widely.

Finally England has a much higher number of live donors than Spain, emphasizing the complexity of organ donation and the fact that there are more ways to increase the number of organs available, reflecting that deceased donor consent rates are not the only measure of a successful organ donation system.

## Conclusion

The Spanish system has a simpler and more streamlined approach to family consent to organ donation and the documents very proactively encourage donation. If England’s ambition is to achieve the consent rates consistently seen in Spain, there may be opportunities to do so by giving greater legal protection and status to the ODR and also by changing the culture from being impartial and risk-averse toward the promotion of organ donation. Significant investment in staff and resources would also be required to match the availability of ICU beds seen in Spain as well as dedicated resources, including specialist sites, which were previously deemed too expensive to invest in. However, there are potentially modifiable issues that appear to work better in Spain such as a shorter and simpler consent process and much more positive language throughout the process, which would improve the experience of staff and acutely bereaved families. In parallel, research is needed (ideally in a controlled context [39]) to understand more about what works, for whom and why in order to maintain the supply of organs to meet the increasing demand.

## Data Availability

The original contributions presented in the study are included in the article/supplementary material, further inquiries can be directed to the corresponding authors.
